# Discovery of ex situ individuals of *Andrias sligoi*, an extremely endangered species and one of the largest amphibians worldwide

**DOI:** 10.1038/s41598-024-52907-6

**Published:** 2024-01-31

**Authors:** Kanto Nishikawa, Masafumi Matsui, Natsuhiko Yoshikawa, Atsushi Tominaga, Koshiro Eto, Ibuki Fukuyama, Kazumi Fukutani, Kohei Matsubara, Yasunari Hattori, Shohei Iwato, Tsukasa Sato, Zenkichi Shimizu, Hirokazu Onuma, Sotaro Hara

**Affiliations:** 1 Graduate School of Global Environmental Studies, Kyoto University, Yoshida Honmachi, Sakyo, Kyoto, 606-8501 Japan; 2 Graduate School of Human and Environmental Studies, Kyoto University, Yoshida Nihonmatsu, Sakyo, Kyoto, 606-8501 Japan; 3 Department of Zoology, National Museum of Nature and Science, Tokyo 4-1-1 Amakubo, Tsukuba, Ibaraki 305-0005 Japan; 4 Faculty of Education, University of the Ryukyus, Senbaru 1, Nishihara, Okinawa 903-0213 Japan; 5 Kitakyushu Museum of Natural History and Human History, 2-4-1 Higashida, Yahatahigashi, Kitakyushu, Fukuoka 805-0071 Japan; 6 Faculty of Integrated Human Studies, Kyoto University, Yoshida Nihonmatsu, Sakyo, Kyoto, 606-8501 Japan; 7 Mie Natural History Research Group, 1386-17 Hioka-cho, Matsusaka, Mie 515-0835 Japan; 8 The Nature Conservation Society of Hyogo Prefecture, 2-1-18-704, Gokoudori, Chuo, Kobe, 651-0087 Japan

**Keywords:** Genetic hybridization, Population genetics, Herpetology

## Abstract

The South China giant salamander, *Andrias sligoi*, is one of the largest extant amphibian species worldwide. It was recently distinguished from another Chinese species, the Chinese giant salamander, *Andrias davidianus*, which is considered Critically Endangered according to the International Union for Conservation of Nature (IUCN) Red List. It appears too late to save this extremely rare and large amphibian in situ. Another extant species of the same genus, *Andrias japonicus*, inhabits Japan. However, the introduction of Chinese giant salamanders into some areas of Japan has resulted in hybridization between the Japanese and Chinese species. During our genetic screening of giant salamanders in Japan, we unexpectedly discovered four individuals of the South China giant salamander: two were adult males in captivity, and one had recently died. The last individual was a preserved specimen. In this study, we report these extremely rare individuals of *A. sligoi* in Japan and discuss the taxonomic and conservational implications of these introduced individuals.

## Introduction

**Figure 1:**
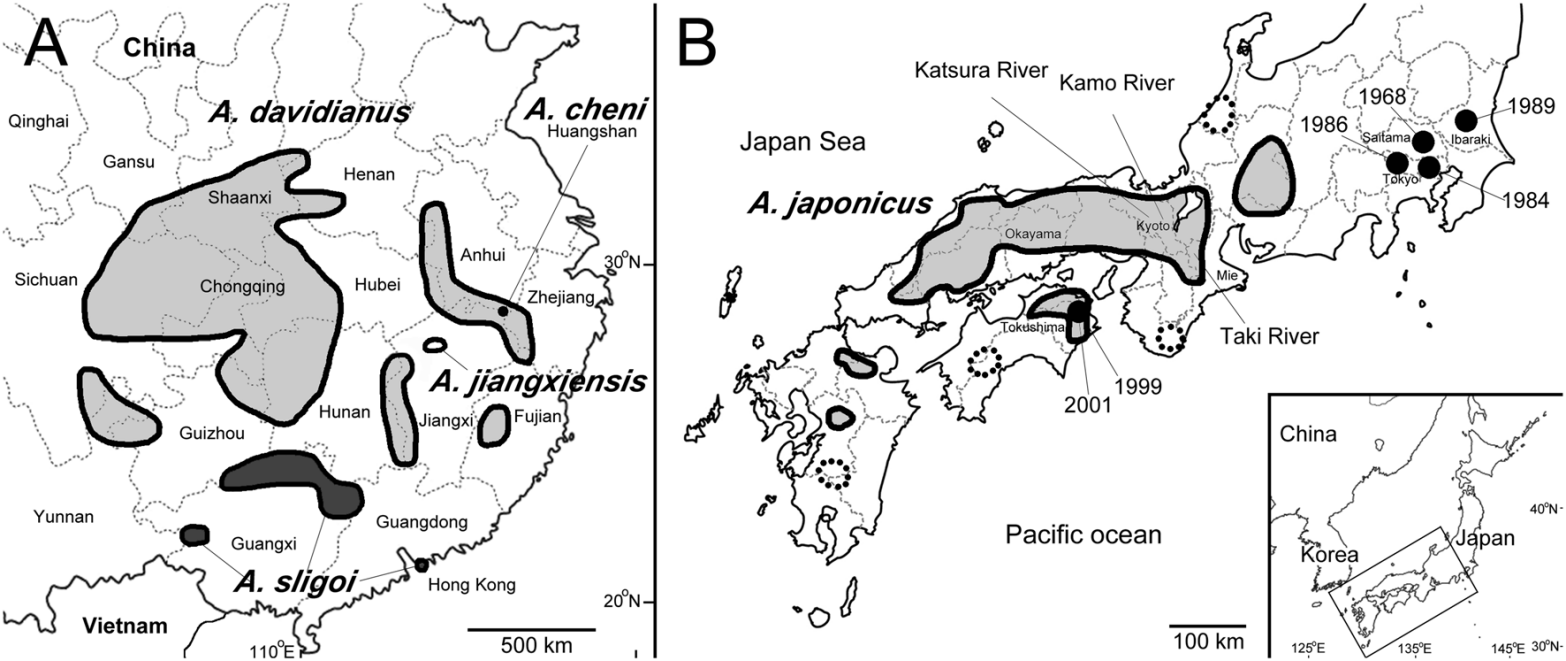
(**A**) Map of eastern China showing the original range of *A. davidianus* (five lightly shaded areas), *A. sligoi* (three darkly shaded areas), and *A. jiangxiensis* (one open circle). The locality of *A. davidianus* in Qinghai Province was omitted in this figure. The ranges were generated based on publications^2,4,8^. (**B**) Map of central and southwestern Japan showing the range of *A. japonicus* (shaded areas). Possible artificial distribution is highlighted by dotted lines. Black circles show collection locations and years of *A. davidianus* sensu stricto in the wild (two individuals were collected from Saitama in 1986). This figure was generated by Adobe Photoshop 2023 (vers. 24.7.0).

Giant salamanders are regarded as the largest extant amphibians worldwide and belong to the family Cryptobranchidae, including *Andrias* and *Cryptobranchus*. All species are endangered^1^ and listed on the appendices of the Convention on International Trade in Endangered Species of Wild Fauna and Flora (CITES). All extant *Andrias* species occur in Japan and China (Fig. 1), and their conservation is an urgent concern, particularly in China^2–4^. Until recently, only the Chinese giant salamander *A. davidianus* (Blanchard 1871) was known in China; wild populations of this species have declined considerably due to environmental destruction and overharvesting for food and traditional medicine^5,6^. Numerous commercial farms must have bred giant salamanders using local individuals, as well as non-local individuals from other provinces, because matured brood stocks are usually difficult to collect near the farms; this approach results in the generation of hybridized individuals between genetically differentiated populations. Reintroduction of artificially bred individuals into the wild was conducted to reinforce wild populations^4^; however, it constituted another threat to the protection of the original genetic diversity and possible cryptic species within *A. davidianus*^7^.

Amid this critical situation in China, the South China giant salamander, *Andrias sligoi* (Boulenger 1924), was resurrected^8^, and the Jiangxi giant salamander, *A. jiangxiensis* Lu, Wang, Chai, Yi, Peng, Murphy, Zhang et Che 2022 and Qimen Giant Salamander, *A. cheni* Xu, Gong, Li, Jiang, Huang et Huang 2023 were newly described. Turvey et al.^8^ recovered almost complete mitochondrial genomes from the holotype of *A. sligoi* collected in 1920 and other museum specimens of Chinese giant salamanders deposited in the late twentieth century before the start of artificial transportation^3^; they assigned these specimens to the lineages of wild-caught individuals. Although original range of *Andrias sligoi* is still controversial, it is presumably the largest amphibian among all extant species worldwide^8^. Unfortunately, when the species was resurrected, it was nearly extinct in the wild because of the situations noted above^8^.

The genus *Andrias* includes another extant species, the Japanese giant salamander *A. japonicus* (Temminck 1836), which was also harvested for food and medicine before World War II. In 1952, the species was legally protected. Nonetheless, the demand for giant salamanders did not decrease, and hundreds of Chinese giant salamanders were imported to Japan as a substitute for the protected native species^9^. These Chinese giant salamanders were released or escaped into Japanese rivers, then interbred with *A. japonicus* and established hybrid swarms in several areas of Japan^10^. To assess the present hybridization situation and to search for living Chinese giant salamanders possibly present in Japan, we conducted genetic surveys. We discovered that the South China giant salamander (believed to be extinct) as well as potentially undescribed species (imperiled in China) present in Japan. Here, we report this unexpected finding, describe the morphology of this rare species for taxonomic implications, and discuss the conservational implications of this invasive but endangered species.

## Results

### Samples

We made field night surveys in the Kamogawa River, Kyoto, Japan from 2007 to 2015 by visual encounter method and collected 68 tissue samples of the giant salamanders. We also collected five tissue samples of *A.* cf. *davidianus* (*A. sligoi* and U1 lineage: see below) from private houses, aquariums, and zoos throughout Japan (Tables 1, 2).

**Table 1:** Samples used for mtDNA analysis in this study together with the information on locality and GenBank accession numbers. Microsatellite identifications are shown in parentheses.

Sample number	Species	Haplotype clades by Yan et al.^4^*	Voucher	Locality	Genbank	Reference
1	*Andrias davidianus*	A	GXXA609	China, Xingan, Guilin, Guangxi	KU131056	^11^
2	*Andrias davidianus*	A	KIZGXDN3	China, Maoershan, Guilin, Guangxi	MH051462	^4^
3	*Andrias davidianus*	A	KIZYPX10536	China, Maoershan, Guilin, Guangxi	MH051461	^4^
4	*A. japonicus* × *A. davidianus* (F1)	B	KUHE 41349	Japan, Kyoto, Kyoto, Kamo River	LC650372	This study
5	*Andrias davidianus* (*A. davidianus*)	B	KUHE 42280	Japan, Kyoto, Kyoto, Kamo River	LC650374	This study
6	*A. japonicas* × *A. davidianus* (Backcross of *A. davidianus*)	B	KUHE 43978	Japan, Kyoto, Kyoto, Kamo River	LC650375	This study
7	*A. japonicus* × *A. davidianus* (Backcross of *A. davidianus*)	B	KUHE 44971	Japan, Kyoto, Kyoto, Kamo River	LC650376	This study
8	*A. japonicus* × *A. davidianus* (F1)	B	KUHE 46398	Japan, Kyoto, Kyoto, Kamo River	LC650377	This study
9	*A. japonicus* × *A. davidianus* (Backcross of *A. davidianus*)	B	KUHE 46434	Japan, Kyoto, Kyoto, Kamo River	LC650378	This study
10	*A. japonicus* × *A. davidianus* (F1)	B	KUHE 46470	Japan, Kyoto, Kyoto, Kamo River	LC650379	This study
11	*Andrias davidianus* (*A. davidianus*)	B	KUHE 46523	Japan, Kyoto, Kyoto, Kamo River	LC650380	This study
12	*A. japonicus* × *A. davidianus* (Backcross of *A. davidianus*)	B	KUHE 48469	Japan, Kyoto, Kyoto, Kamo River	LC650381	This study
13	*A. japonicus* × *A. davidianus* (Backcross of *A. davidianus*)	B	KUHE 48479	Japan, Kyoto, Kyoto, Kamo River	LC650382	This study
14	*A. japonicus* × *A. davidianus* (Backcross of *A. davidianus*)	B	KUHE 48480	Japan, Kyoto, Kyoto, Kamo River	LC650383	This study
15	*A. japonicus* × *A. davidianus* (F1)	B	KUHE 55134	Japan, Kyoto, Kyoto, Kamo River	LC650384	This study
16	*A. japonicus* × *A. davidianus* (others)	B	KUHE 56198	Japan, Kyoto, Kyoto, Kamo River	LC650386	This study
17	*A. japonicus* × *A. davidianus* (Backcross of *A. davidianus*)	B	KUHE 56599	Japan, Kyoto, Kyoto, Kamo River	LC650387	This study
18	*A. japonicus* × *A. davidianus* (others)	B	KUHE 56826	Japan, Kyoto, Kyoto, Kamo River	LC650388	This study
19	*A. japonicus* × *A. davidianus* (others)	B	KUHE 56887	Japan, Kyoto, Kyoto, Kamo River	LC650389	This study
20	*A. japonicus* × *A. davidianus* (Backcross of *A. davidianus*)	B	KUHE 57676	Japan, Kyoto, Kyoto, Kamo River	LC650390	This study
21	*Andrias davidianus* (*A. davidianus*)	B	KUHE 57681	Japan, Kyoto, Kyoto, Kamo River	LC650391	This study
22	*A. japonicus* × *A. davidianus* (F1)	B	KUHE 58629	Japan, Kyoto, Kyoto, Kamo River	LC650392	This study
23	*A. japonicus* × *A. davidianus* (F1)	B	KUHE 58631	Japan, Kyoto, Kyoto, Kamo River	LC650393	This study
24	*A. japonicus* × *A. davidianus* (F1)	B	KUHE 58639	Japan, Kyoto, Kyoto, Kamo River	LC650394	This study
25	*A. japonicus* × *A. davidianus* (F1)	B	KUHE 58640	Japan, Kyoto, Kyoto, Kamo River	LC650395	This study
26	*A. japonicus* × *A. davidianus* (F1)	B	KUHE 58675	Japan, Kyoto, Kyoto, Kamo River	LC650396	This study
27	*A. japonicus* × *A. davidianus* (others)	B	KUHE 58678	Japan, Kyoto, Kyoto, Kamo River	LC650397	This study
28	*A. japonicus* × *A. davidianus* (others)	B	KUHE 58687	Japan, Kyoto, Kyoto, Kamo River	LC650398	This study
29	*A. japonicus* × *A. davidianus* (Backcross of *A. davidianus*)	B	KUHE 58774	Japan, Kyoto, Kyoto, Kamo River	LC650400	This study
30	*A. japonicus* × *A. davidianus* (F1)	B	KUHE 58785	Japan, Kyoto, Kyoto, Kamo River	LC650401	This study
31	*A. japonicus* × *A. davidianus* (Backcross of *A. davidianus*)	B	KUHE 58899	Japan, Kyoto, Kyoto, Kamo River	LC650402	This study
32	*Andrias davidianus* (*A. davidianus*)	B	KUHE 58901	Japan, Kyoto, Kyoto, Kamo River	LC650403	This study
33	*A. japonicus* × *A. davidianus* (Backcross of *A. davidianus*)	B	KUHE 58905	Japan, Kyoto, Kyoto, Kamo River	LC650404	This study
34	*A. japonicus* × *A. davidianus* (others)	B	KUHE 58918	Japan, Kyoto, Kyoto, Kamo River	LC650406	This study
35	*A. japonicus* × *A. davidianus* (F1)	B	KUHE 58927	Japan, Kyoto, Kyoto, Kamo River	LC650407	This study
36	*A. japonicus* × *A. davidianus* (Backcross of *A. davidianus*)	B	KUHE 59014	Japan, Kyoto, Kyoto, Kamo River	LC650408	This study
37	*A. japonicus* × *A. davidianus* (F1)	B	KUHE 59085	Japan, Kyoto, Kyoto, Katsura River	LC650409	This study
38	*A. japonicus* × *A. davidianus* (Backcross of *A. davidianus*)	B	KUHE 62129	Japan, Kyoto, Kyoto, Kamo River	LC650410	This study
39	*A. japonicus* × *A. davidianus* (Backcross of *A. davidianus*)	B	KUHE no number	Japan, Kyoto, Kyoto, Kamo River	LC650411	This study
40	*A. japonicus* × *A. davidianus* (F1)	B	KUHE no number	Japan, Kyoto, Kyoto, Kamo River	LC650412	This study
41	*Andrias davidianus* (*A. davidianus*)	B	KUHE no number	Japan, Kyoto, Kyoto, Kamo River	LC650413	This study
42	*A. japonicus* × *A. davidianus* (Backcross of *A. davidianus*)	B	KUHE no number	Japan, Kyoto, Kyoto, Kamo River	LC650414	This study
43	*A. japonicus* × *A. davidianus* (Backcross of *A. davidianus*)	B	KUHE no number	Japan, Kyoto, Kyoto, Kamo River	LC650415	This study
44	*A. japonicus* × *A. davidianus* (Backcross of *A. davidianus*)	B	KUHE no number	Japan, Kyoto, Kyoto, Kamo River	LC650416	This study
45	*A. japonicus* × *A. davidianus* (Backcross of *A. davidianus*)	B	KUHE no number	Japan, Kyoto, Kyoto, Kamo River	LC650417	This study
46	*A. japonicus* × *A. davidianus* (Backcross of *A. davidianus*)	B	KUHE no number	Japan, Kyoto, Kyoto, Kamo River	LC650418	This study
47	*A. japonicus* × *A. davidianus* (Backcross of *A. davidianus*)	B	KUHE no number	Japan, Kyoto, Kyoto, Kamo River	LC650419	This study
48	*A. japonicus* × *A. davidianus* (others)	B	KUHE no number	Japan, Kyoto, Kyoto, Kamo River	LC650420	This study
49	*A. japonicus* × *A. davidianus* (Backcross of *A. davidianus*)	B	KUHE no number	Japan, Kyoto, Kyoto, Kamo River	LC650421	This study
50	*Andrias davidianus* (*A. davidianus*)	B	KUHE no number	Japan, Kyoto, Kyoto, Kamo River	LC650422	This study
51	*A. japonicus* × *A. davidianus* (Backcross of *A. davidianus*)	B	No voucher	Japan, Kyoto, Kyoto, Kamo River	LC650425	This study
52	*A. japonicus* × *A. davidianus* (F1)	B	No voucher	Japan, Kyoto, Kyoto, Kamo River	LC650426	This study
53	*A. japonicus* × *A. davidianus* (others)	B	No voucher	Japan, Kyoto, Kyoto, Kamo River	LC650427	This study
54	*Andrias davidianus*	B	HNJY390	China, Wangwoshan, Jiyuan, Henan	KU131048	^11^
55	*Andrias davidianus*	B	HNSZSDJ82	China, Yuanzi Cave, Shangdongjie, Sangzhi, Zhangjiajie, Hunan	KU131061	^11^
56	*Andrias davidianus*	B	KIZYPX25999	China, Qingchuan, Guangyuan, Sichuan	MH051426	^4^
57	*Andrias davidianus*	B	KIZYPX44113	China, Lushi, Henan	MH051424	^4^
58	*Andrias davidianus* (Reference)	B	KUHE 34380	Unknown locality in China	AB445782	^14^
59	*Andrias davidianus* (*A. davidianus*)	B	KUHE 41271	Japan, Kyoto, Kyoto, Kamo River	LC650428	This study
60	*Andrias davidianus* (*A. davidianus*)	B	KUHE 47982	Japan, Kyoto, Kyoto, Kamo River	LC650429	This study
61	*Andrias davidianus* (*A. davidianus*)	B	KUHE 48474	Japan, Kyoto, Kyoto, Kamo River	LC650430	This study
62	*A. japonicus* × *A. davidianus* (others)	B	KUHE 55714	Japan, Kyoto, Kyoto, Kamo River	LC650431	This study
63	*Andrias davidianus* (*A. davidianus*)	B	KUHE 56189	Japan, Kyoto, Kyoto, Kamo River	LC650432	This study
64	*Andrias davidianus* (*A. davidianus*)	B	KUHE 56335	Japan, Kyoto, Kyoto, Kamo River	LC650433	This study
65	*Andrias davidianus* (*A. davidianus*)	B	KUHE 56829	Japan, Kyoto, Kyoto, Kamo River	LC650434	This study
66	*Andrias davidianus* (*A. davidianus*)	B	KUHE 58902	Japan, Kyoto, Kyoto, Kamo River	LC650435	This study
67	*Andrias davidianus* (*A. davidianus*)	B	KUHE 59308	Japan, Kyoto, Kyoto, Kamo River	LC650437	This study
68	*Andrias davidianus* (*A. davidianus*)	B	KUHE 62130	Japan, Kyoto, Kyoto, Kamo River	LC650438	This study
69	*Andrias davidianus* (*A. davidianus*)	B	KUHE no number	Japan, Kyoto, Kyoto, Kamo River	LC650441	This study
70	*Andrias davidianus* (*A. davidianus*)	B	KUHE no number	Japan, Kyoto, Kyoto, Kamo River	LC650442	This study
71	*Andrias davidianus* (*A. davidianus*)	B	KUHE no number	Japan, Kyoto, Kyoto, Kamo River	LC650443	This study
72	*Andrias davidianus* (Reference)	B	No voucher	Unknown locality in China	LC650446	This study
73	*Andrias davidianus* (*A. davidianus*)	B	No voucher	Japan, Kyoto, Kyoto, Kamo River	LC650447	This study
74	*Andrias davidianus* (*A. davidianus*)	B	No voucher	Japan, Kyoto, Kyoto, Kamo River	LC650448	This study
75	*Andrias davidianus*	B	SCMB244	China, Mabian, Leshan, Sichuan	KU131043	^29^
76	*Andrias davidianus*	B	YNYL551	China, Niujie, Yiliang, Zhaotong, Yunnan	KU131053	^29^
77	*Andrias davidianus* (*A. davidianus*)	B	KUHE 56597	Japan, Kyoto, Kyoto, Kamo River	LC650449	This study
78	*Andrias davidianus* (*A. davidianus*)	B	KUHE 56600	Japan, Kyoto, Kyoto, Kamo River	LC650450	This study
79	*Andrias davidianus* (*A. davidianus*)	B	KUHE no number	Japan, Kyoto, Kyoto, Kamo River	LC650451	This study
80	*Andrias davidianus*	C	KIZYPX25990	China, Qingchuan, Guangyuan, Sichuan	MH051427	^4^
81	*Andrias davidianus*	C	KIZYPX25991	China, Qingchuan, Guangyuan, Sichuan	MH051428	^4^
82	*Andrias sligoi*	D	CQWL481	China, Wujiang, Yangzte R	KU131051	^29^
83	*Andrias sligoi*	D	GZGDYX583	China, Xiyejing Cave, Yanxia, Guiding, Qiannan, Guizhou	KU131054	^29^
84	*Andrias sligoi*	D	HNLS55	China, Mengdonghe, Yuanjiang, Yangzte R	KU131052	^29^
85	*Andrias sligoi*	D	HNWMY48	China, Wumuyu Cave, Yongding, Zhangjiajie, Hunan	KU131050	^29^
86	*Andrias sligoi*	D	KIZYPX2513	China, Xinglong, Chongqing	MH051435	^4^
87	*Andrias sligoi*	D	KIZZA2	China, Zhengan, Zunyi, Guizhou	MH051442	^4^
88	*Andrias sligoi*	D	KIZZA9	China, Zhengan, Zunyi, Guizhou	MH051443	^4^
89	*Andrias sligoi*	D	No voucher	Unknown locality in China	LC650452	This study
90	*Andrias sligoi*	D	No voucher	Unknown locality in China	LC650453	This study
91	*Andrias sligoi*	D	No voucher	Unknown locality in China	LC650454	This study
92	*Andrias sligoi*	D	KUHE 41444	Unknown locality in China	LC728249	This study
93	*Andrias sligoi*	D	ROM11041	China, Xi'an, Shaanxi, Yellow River	MK177470	^8^
94	*Andrias sligoi*	D	Unknown	unknown	NC004926	^30^
95	*Andrias sligoi*	D	ZMB24105	China, Guangdong or Guangxi, Pearl River	MK177465	^8^
96	*Andrias davidianus*	E	AHHS695	China, Liukou, Xiuning, Huangshan, Anhui	KU131060	^29^
97	*Andrias davidianus*	E	KIZYPX6151	China, Huangshan, Anhui	MH051473	^4^
98	*Andrias davidianus*	E	KIZYPX6152	China, Huangshan, Anhui	MH051474	^4^
99	*Andrias davidianus*	E	ZJLSQY680	China, Xianliang Cave, Qingyuan, Lishui, Zhejiang	KU131059	^29^
100	*Andrias davidianus*	U1	CGS1009	China, Farm-bred (Guangxi)	MH051478	^4^
101	*Andrias davidianus*	U1	CGS725	China, Farm-bred (Jiangxi)	MH051480	^4^
102	*Andrias davidianus*	U1	GXZY587	China, Zishui, Yangzte R	KU131055	^29^
103	*Andrias davidianus*	U1	KUHE 65273	Japan, Tokushima, Komatsushima, Tatsue River	AB445784	^14^
104	*Andrias jiangxiensis*	U2	CGS291	China, Farm-bred (Jiangxi)	MH051481	^4^
105	*Andrias jiangxiensis*	U2	GDLZ365	China, Lianzhou, Qingyuan, Guangdong	KU131046	^29^
106	*Andrias jiangxiensis*	U2	JXJA336	China, Jingan, Yichuan, Jiangxi	KU131044	^29^
107	*Andrias jiangxiensis*	U2	JXJGS352	China, Maoping, Jinggangshan, Jian, Jiangxi	KU131045	^29^
108	*Andrias japonicus*		No voucher	Japan, Kumamoto, Asagiri, Kuma River	AB445780	^14^
109	*Cryptobranchus alleganiensis*		No voucher	unknown	GQ368662	^31^

Institutional abbreviations: *KUHE* Graduate School of Human and Environmental Studies, Kyoto University, *ROM* Royal Ontario Museum, Toronto, *ZMB* Museum für Naturkunde, Berlin (original names of remaining abbreviations are unkown in the sources).

**Table 2:** Samples used for microsatellite analysis in this study.

Sample number	Species	Voucher/PIT tag number	Locality	
1	*Andrias davidianus*	KUHE 34378	Unknown locality in China	
2	*Andrias davidianus*	KUHE 34380	Unknown locality in China	
3	*Andrias davidianus*	KUHE 41271	Japan, Kyoto, Kyoto, Kamo River	
4	*Andrias davidianus*	KUHE 42280	Japan, Kyoto, Kyoto, Kamo River	
5	*Andrias davidianus*	KUHE 46523	Japan, Kyoto, Kyoto, Kamo River	
6	*Andrias davidianus*	KUHE 47982	Japan, Kyoto, Kyoto, Kamo River	
7	*Andrias davidianus*	KUHE 48474	Japan, Kyoto, Kyoto, Kamo River	
8	*Andrias davidianus*	KUHE 56189	Japan, Kyoto, Kyoto, Kamo River	
9	*Andrias davidianus*	KUHE 56335	Japan, Kyoto, Kyoto, Kamo River	
10	*Andrias davidianus*	KUHE 56597	Japan, Kyoto, Kyoto, Kamo River	
11	*Andrias davidianus*	KUHE 56600	Japan, Kyoto, Kyoto, Kamo River	
12	*Andrias davidianus*	KUHE 56829	Japan, Kyoto, Kyoto, Kamo River	
13	*Andrias davidianus*	KUHE 57681	Japan, Kyoto, Kyoto, Kamo River	
14	*Andrias davidianus*	KUHE 58901	Japan, Kyoto, Kyoto, Kamo River	
15	*Andrias davidianus*	KUHE 58902	Japan, Kyoto, Kyoto, Kamo River	
16	*Andrias davidianus*	KUHE 59308	Japan, Kyoto, Kyoto, Kamo River	
17	*Andrias davidianus*	KUHE 62130	Japan, Kyoto, Kyoto, Kamo River	
18	*Andrias davidianus*	KUHE no number	Japan, Kyoto, Kyoto, Kamo River	
19	*Andrias davidianus*	KUHE no number	Japan, Kyoto, Kyoto, Kamo River	
20	*Andrias davidianus*	KUHE no number	Japan, Kyoto, Kyoto, Kamo River	
21	*Andrias davidianus*	KUHE no number	Japan, Kyoto, Kyoto, Kamo River	
22	*Andrias davidianus*	KUHE no number	Japan, Kyoto, Kyoto, Kamo River	
23	*Andrias davidianus*	KUHE no number	Japan, Kyoto, Kyoto, Kamo River	
24	*Andrias davidianus*	No voucher	Unknown locality in China	
25	*Andrias davidianus*	No voucher	Japan, Kyoto, Kyoto, Kamo River	
26	*Andrias davidianus*	No voucher	Japan, Kyoto, Kyoto, Kamo River	
27	*Andrias davidianus*	No voucher	Unknown locality in China	
28	*Andrias davidianus*	No voucher	Unknown locality in China	
29	*Andrias davidianus*	No voucher	Unknown locality in China	
30	*Andrias davidianus*	No voucher	Unknown locality in China	
31	*Andrias japonicus*	392145000063723	Japan, Nara, Fukatani River	
32	*Andrias japonicus*	00075BBD7E	Japan, Nara, Muro River	
33	*Andrias japonicus*	00075BED8C	Japan, Nara, Fukatani River	
34	*Andrias japonicus*	00075C28CE	Japan, Nara, Muro River	
35	*Andrias japonicus*	00075C2DEE	Japan, Nara, Fukatani River	
36	*Andrias japonicus*	00075C2E6B	Japan, Nara, Nagatani River	
37	*Andrias japonicus*	00075C3978	Japan, Nara, Nagatani River	
38	*Andrias japonicus*	00075C83E1	Japan, Mie, Muro River	
39	*Andrias japonicus*	00075CB299	Japan, Mie, Ashouzu River	
40	*Andrias japonicus*	0006B846CB	Japan, Kyoto, Kyoto, Kiyotaki River	
41	*Andrias japonicus*	0006B84754	Japan, Kyoto, Kyoto, Kiyotaki River	
42	*Andrias japonicus*	0006B84E8D	Japan, Kyoto, Kyoto, Katsura River	
43	*Andrias japonicus*	No voucher	Japan, Mie, Maefukase River	
44	*Andrias japonicus*	No voucher	Japan, Mie, Maefukase River	
45	*Andrias japonicus*	No voucher	Japan, Mie, Maefukase River	
46	*Andrias japonicus*	No voucher	Japan, Mie, Maefukase River	
47	*Andrias japonicus*	No voucher	Japan, Mie, Maefukase River	
48	*Andrias japonicus*	No voucher	Japan, Mie, Maefukase River	
49	*Andrias japonicus*	No voucher	Japan, Mie, Maefukase River	
50	*Andrias japonicus*	No voucher	Japan, Mie, Maefukase River	
51	*Andrias japonicus*	No voucher	Japan, Mie, Maefukase River	
52	*Andrias japonicus*	No voucher	Japan, Mie, Maefukase River	
53	*Andrias japonicus*	No voucher	Japan, Mie, Maefukase River	
54	*Andrias sligoi*	KUHE 41444	Unknown locality in China	
55	*Andrias sligoi*	No voucher	Unknown locality in China	
56	*Andrias sligoi*	No voucher	Unknown locality in China	
57	*Andrias sligoi*	No voucher	Unknown locality in China	
58	*Andrias davidianus* (U1)	KUHE 65273	Japan, Tokushima, Komatsushima, Tatsue River	

Institutional abbreviations: *KUHE* Graduate School of Human and Environmental Studies, Kyoto University.

### mtDNA phylogenetic analysis

**Figure 2:**
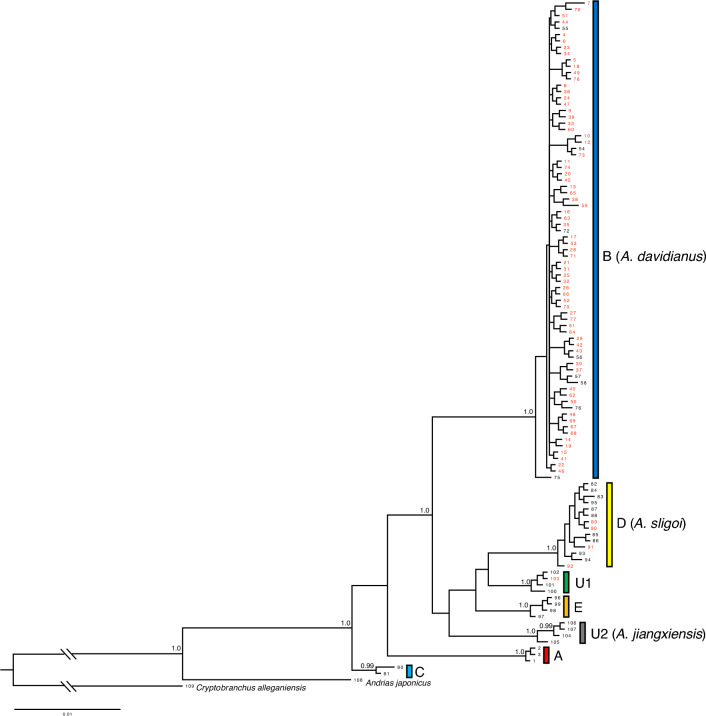
Bayesian phylogenetic reconstruction of partial cyt b gene sequences from giant salamanders. Lineage assignment was in accordance with Yan et al.^4^. Red-colored samples were discovered in Japan. Numbers on nodes indicate significant supports (BI ≥ 0.95). For more details regarding sample numbers, see Table 1.

**Figure 3:**
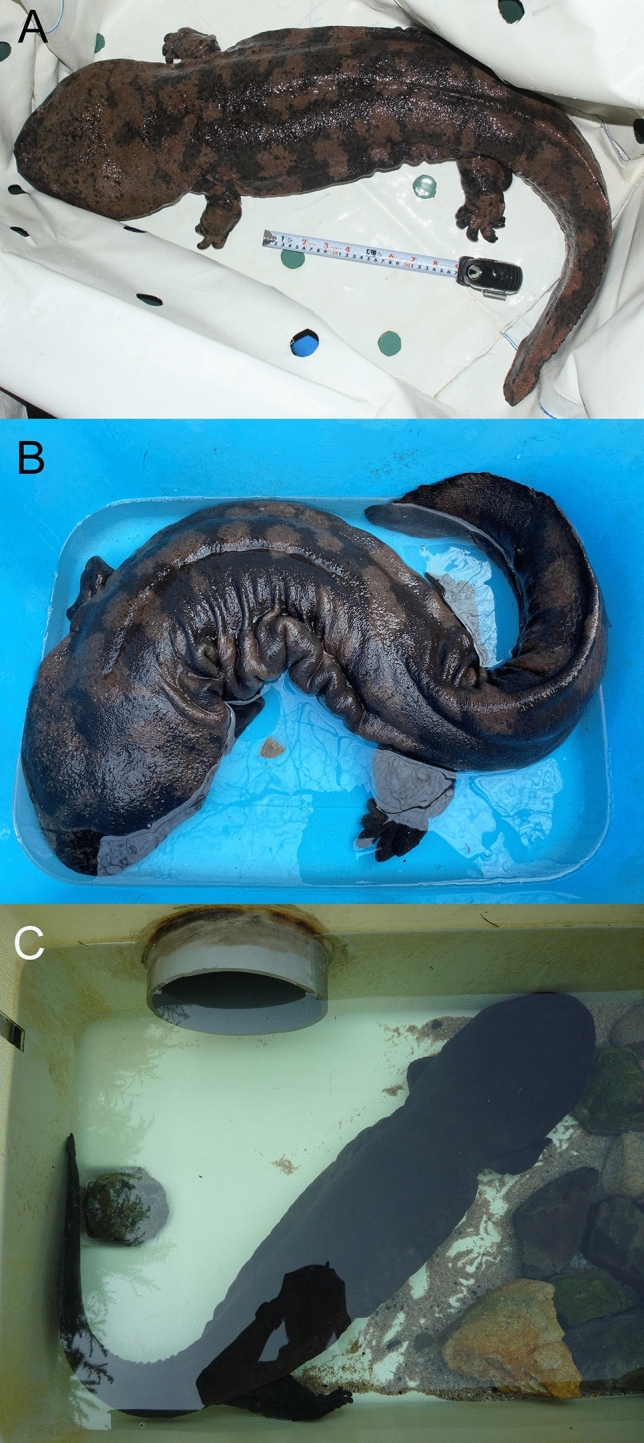
Photos of *A. sligoi* in Japan. (**A**) An individual presently housed at the Sunshine Aquarium, Tokyo, Japan (photo acquired on 25 March 2020). (**B**) An individual presently housed at the Hiroshima City Asa Zoological Park (photo acquired on 23 December 2021. Copyright: Hiroshima City Asa Zoological Park). (**C**) A deceased individual once kept in a private house in Okayama Prefecture (photo acquired on 21 June 2011).

The BI tree obtained based on 590–1141 bp mtDNA cyt b sequences (Fig. 2). The 73 samples collected in this study consisted of three of the seven Chinese mitochondrial lineages reported^4^: lineages B, D, and U1. Four individuals recovered within lineage D, which is assigned the critically endangered species *A. sligoi* (Table 1, Fig. 3). Among the three Chinese lineages recovered, lineage B was predominant (Table 1). This lineage is naturally distributed in the Yellow River and Yangtze River drainages. Yan et al.^4^ reported that the two lineages U1 and U2 were exclusively found in Chinese farms. However, lineage U2 was recently described as *A. jiangxiensis* based on wild populations. In the present study, one sample collected from Tokushima Prefecture (Sample 103 in Table 1) was assigned to lineage U1.

Uncorrected p-distances using 810–1141 bp between lineages ranged from 1.4–1.7% (*A. sligoi* vs. U1 and lineage E vs. U1) to 3.5–4.0% (lineage A vs. U2) (Supplementary information 1***). The genetic distance between *A. davidianus* (lineage B) and *A. sligoi* (lineage D) was 2.6–3.2%. The distance between *A. japonicus* and *A. davidianus* (lineage B) was 6.0–6.2%.

### Microsatellite genotyping

We successfully obtained genotyping data of 14 microsatellite loci for all 68 samples examined, including 45 hybrids and 23 *A. davidianus* sensu lato. Among the hybrids, 14 were F1, 22 were backcrossed to each parental species, and nine were classified as “other hybrids” whose ancestries are ambiguous. We found no F2 hybrids (Table 2).

**Figure 4:**
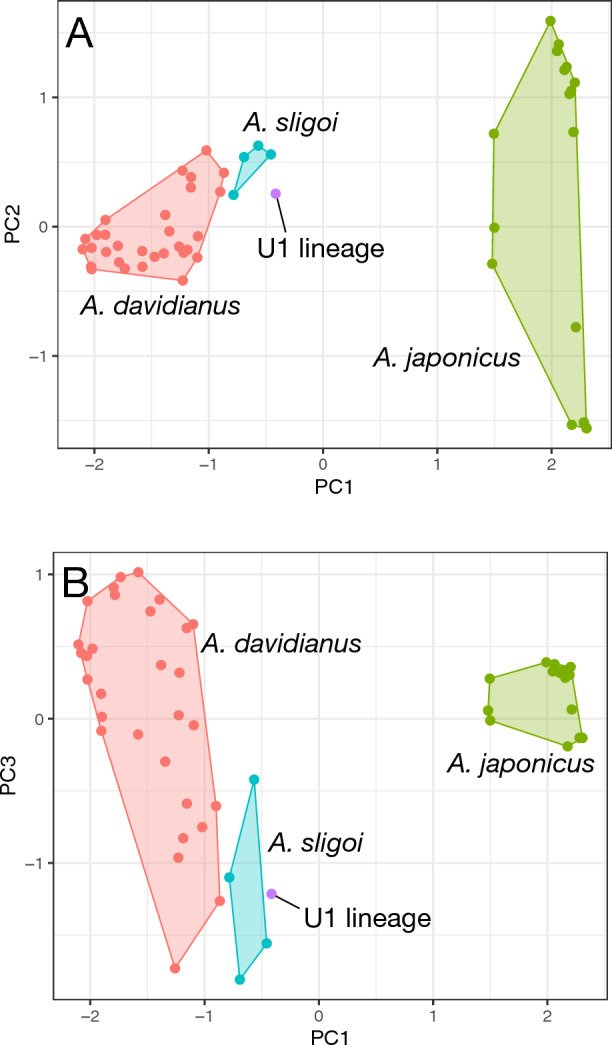
First and second axes (**A**) and first and third axes (**B**) of the PCA plot based on genotype information obtained from microsatellite analyses. *A. davidianus*: red circles, *A. sligoi*: blue circles, U1 lineage: purple circle, *A. japonicus*: green circles.

The PCA plot based on 14 microsatellite loci of 58 individuals (including 23 *A. davidianus* from Kamogawa River [identified by NewHybrids,] seven *A. davidianus* in captivity, 23 *A. japonicus* from Kyoto, Mie, and Nara prefectures, four *A. sligoi* [identified by mtDNA analysis], and one U1 lineage [identified by mtDNA analysis]) revealed two main groups separated along the first axis (43.6%): (1) *A. japonicus* and (2) *A. davidianus*, *A. sligoi*, and U1 lineage, which were identified by mtDNA analysis (Fig. 2). Although *A. sligoi* and U1 lineage were slightly separated from *A. davidianus* in the first axis, they largely overlapped in the second (9.2%) and third (6.2%) axes (Fig. 4A,B).

### Sex determination

The two female specimens of *A. davidianus* showed the expected female-specific bands in the four genetic markers associated with the W sex-chromosome^11^, whereas the two male specimens did not show any band. These results validate the sexual identification method used in this study. Two living *A. sligoi* specimens did not show any band and were identified as males; of the other two specimens, the first had died and was deposited as a voucher (KUHE 41444) while the second died and no voucher was kept.

### Morphological description of *A. sligoi*

All four individuals examined (including specimen KUHE 41444 from the museum collection) exhibited a robust and large body structure, as well as a wide and flat head with small dorsal and lateral tubercles (absent on the ventral side) including some paired tubercles. The skin was smooth; cutaneous folds on the lateral body were thick and well-developed. The tail was shorter than the snout-vent length. The body color was highly contrasted, with dark and pale brown colors (Fig. 3A,B). One individual, which recently died, was completely black (Fig. 3C).

One of the specimens, a preserved, large adult female at Kyoto University (KUHE 41444, total length = 1115 mm), had the following ratios of each character to the snout-vent length (698 mm): head length: 32% (223 mm); maximum head width: 32% (226 mm); mouth width: 19% (130 mm); lower jaw length: 15% (104 mm); snout length: 9% (61 mm); internarial distance: 5% (38 mm); interorbital distance: 13% (91 mm); vomerine tooth series width: 10% (68 mm); axilla-groin distance: 53% (370 mm); tail length: 60% (417 mm); medial tail height: 17% (121 mm); medial tail width: 5% (36 mm); forelimb length: 21% (144 mm); hindlimb length: 25% (144 mm); first finger length: 2% (16 mm); second finger length: 4% (30 mm); third finger length: 4% (30 mm); fourth finger length: 4% (25 mm); first toe length: 3% (20 mm); second toe length: 5% (36 mm); third toe length: 6% (42 mm); fourth toe length: 5% (33 mm); and fifth toe length: 4% (27 mm). The finger length and toe length formulae were II = III > IV > I and III > II > IV > V > I, respectively. The specimen had no tubercles from the frontal to parietal regions, and tubercles were lined on the ventral surface of its throat.

The illustration of the type specimen of *A. sligoi* (Fig. 2 in the work by Turvey et al.^8^, middle and bottom) shows a typical flat snout similar to the other Chinese species *A. davidianus* and *A. jiangxiensis*, with reddish-brown color and only some small black markings on the ventral side; these findings differ from the highly contrasted or monochromatic black coloration observed in the present study (Fig. 4). The color pattern of the illustration of *A. japonicus* (Fig. 2 in the work by Turvey et al.^8^, top left) is similar to *A. sligoi* in our study, as well as the type specimens (Fig. 2 in the work by Turvey et al.^8^, right). However, the thick head shape in the profile and distinct large tubercles on the head are similar to *A. japonicus*. The type specimen of *A. sligoi* had small tubercles on the head, as described by Boulenger^12^, consistent with observations in the individuals from Japan.

## Discussion

In the genus *Andrias*, hybridization within and between species have occurred both intentionally and accidentally. In this study, most of the non-native individuals found in Japan were hybrids between *A. davidianus* and *A. japonicus*, as well as their backcrosses; some pure Chinese giant salamander species were also discovered in this study. These individuals belonged to three of the seven known mitochondrial *Andrias* lineages, including lineage B corresponding to *A. davidianus*, lineage D corresponding to *A. sligoi*, and lineage U1 corresponding to an unknown population found at Chinese farms. These results suggest that the Chinese giant salamanders exported to Japan were collected from multiple locations in China.

Four individuals were identified as *A. sligoi* via mtDNA analysis, and we determined that they had been imported to Japan in the 1970s to 1980s judging from the information on newspapers at the time^10^ prior to the start of captive breeding in 1994 in Germany^13^ and China^3^, and the release of farmed individuals including hybrids after 2008^4^. Given the information above, these individuals are too large in size (total lengths of the four salamanders are 1100, 1115, 1250, and 1375 mm) to be potential hybrids raised in China. Further, no hybrids have been found in our genetic analyses from available imported Chinese individuals so far. Therefore, we concluded that those four individuals were genetically pure *A. sligoi* originally collected in China. Morphological examination also supported this identification.

Chinese giant salamander populations have experienced a rapid decline since the 1970s because of extensive collection from the wild^2^, which supports our estimated time range of importation and hybridization in Japan. Chinese giant salamander individuals have not been imported to Japan since the 1990s. In Japan, Chinese giant salamander adults currently have minimal likelihood of reproducing with conspecific Chinese individuals and must have decreased in number over time, making them more likely to reproduce with *A. japonicus* and hybrid individuals, than conspecifics. No wild F1 individuals have been observed in monthly surveys since 2011, indicating that the adults of pure Chinese giant salamanders (including *A. sligoi*) are nearly extinct in Japan. *Andrias sligoi* is also nearly extinct in China and will soon disappear, even in the introduced refugia of non-original habitats in Japan.

By this study, the four living *A. sligoi* were discovered in Japan, but the two of them already died. Now the two males are alive in captivity. In 1972, 800 Chinese giant salamander individuals (at least including *A. davidianus* and *A. sligoi*) were imported and kept in artificial ponds in a private house in Okayama Prefecture, but 300 of them died within 1 year according to The Asahi Shimbun newspaper on 28 September 1973. Some of the *A. sligoi* that we discovered in this study may be a part of these remained individuals. One of the two extant *A. sligoi* was bought from a pet shop in Japan on 25 February 1999, with no information regarding the year of import, but it was likely around 20–30 years ago. This individual is now kept in the Sunshine Aquarium in Tokyo, with a total length of approximately 1250 mm, measured on 25 March 2020. The other male was one of 20 individuals illegally imported from Taiwan to Japan and seized at Osaka International Airport on 13 June 1986. It was kept in the Himeji City Aquarium, and its total length and body weight were measured three times: on 2 August 1986 (305 mm and 113.8 g), on 20 April 1996 (890 mm and 4.6 kg), and on 11 December 2008 (1220 mm and 12.4 kg). The male was eventually transferred to the Hiroshima City Asa Zoological Park in 2008, where it currently measures 1375 mm in total length and weighs 23.4 kg (measured on 23 December 2021). These introduced individuals could be ex situ refugia for critically endangered species. Both individuals were genetically identified as male, and their life spans are near the maximum limit of approximately 60 years (our unpublished data). Therefore, we plan to store their sperm and germ cells in the Frozen Zoo of the National Institute for Environmental Studies, Japan, for future artificial reproduction. We are urgently searching for remaining Chinese giant salamander species, in China and elsewhere; we are keeping candidate individuals, particularly females, for captive breeding and future reproduction, with closely collaborating to international amphibian conservation acts including the amphibian ark project (https://www.amphibianark.org/). Time is running out to save the world largest extant amphibian species; international collaboration and action are needed to locate and protect this endangered species.

The discovery of *A. sligoi* individuals has enabled the morphological examination of living individuals by scientists for the first time since its description. The presumed type specimen and its illustration (Fig. 2 in the work by Turvey et al.^8^, left) are useful for understanding the morphological characteristics. However, the illustrator may have incorrectly depicted the color pattern of *A. japonicus* instead of *A. sligoi*, based on our comparisons among the illustrations and the living *A. sligoi* individuals obtained in this study. Further examinations of specimen morphology remain necessary to clarify the species identification.

The present study revealed a small genetic difference between *A. davidianus* and *A. sligoi* in the nuclear genome (Fig. 4) and reconfirmed a small difference in the partial cyt b gene (2.0–3.2%; extended data). The difference in mtDNA is slightly greater than the difference between western and eastern populations of *A. japonicus* (1.2–1.5% (1.3% on average)^14^). Murphy et al.^7^ demonstrated an average allozyme difference of 0.07 between the Pearl River population (Fuchuan; corresponding to *A. sligoi*) and populations of the Yellow and Yangtze Rivers (Chang’an, Yuanqu, and Dayong; corresponding to *A. davidianus* sensu lato except for the Huangshan lineage), based on Nei’s genetic distance. This value is also small at the intraspecific level in salamanders (minimum 0.15 between species^15^), as revealed by our microsatellite result (Fig. 4). Although the genetic differences in giant salamanders tend to be small because of their delayed sexual maturation and longevity^8^, all available genetic results revealed a small difference between *A. davidianus* and *A. sligoi*.

It is inappropriate to conclude that an allopatric population represents a different species solely based on the genetic distance calculated using mtDNA markers. This issue can be addressed by identifying nuclear markers to examine the boundaries of these populations^16^. If the populations are genetically isolated and represent different species, we would expect to see a drastic transition in genetic composition in and around the contact zone. Otherwise, the transition would be gradual and follow the pattern predicted by the isolation by distance model^17^. However, *A. sligoi* was originally described based on an individual that was apparently an escaped captive from a botanical garden in Hong Kong (originally found in a ditch in Hong Kong after heavy rainfall^12^), a region where native giant salamanders are not known to occur. Although the current distribution of *A. sligoi* is presumably in southern China (Fig. 1A), its precise range is unknown; the genetic composition of southern populations of *A. davidianus* sensu lato has been obscured by human-mediated transportation^7^. Additionally, it is difficult to amplify nuclear sequences from historical specimens stored in museums^8^. Considering these circumstances, it is challenging to re-evaluate the taxonomic status of *A. sligoi* using specimens from unaltered localities and additional data of morphology and nuclear markers. All of the morphological characteristics of *A. sligoi* examined in this study are similar to the characteristics of *A. davidianus* and *A. jiangxiensis*. The present study revealed that the external morphologies of living *A. sligoi* individuals are similar to the morphologies of common living and voucher specimens of *A. davidianus* that we observed and examined in Chinese zoos, farms, and museums. This finding contradicts Boulenger’s note^12^ regarding significant differences in morphology between the two species. However, Boulenger described *A. sligoi* based on the difference from *Megalobatrachus maximus* (Tschudi 1837), which at that time was believed to include both *A. davidianus* and *A. japonicus*. Therefore, Boulenger might have only examined *A. japonicus* for comparison, suggesting that there have been no comparisons of populations of *A. sligoi* and *A. davidianus* for taxonomic validity based on morphology.

Although taxonomic revision of these species is a high priority for the clarification of conservation units, urgent action is needed to protect their genetic diversity. Unfortunately an individual of the lineage U1 detected in Japan by this study died recently. In both China and Japan, artificial transportation must be stopped, and genetically pure populations of these species must be protected.

## Online methods

### Samples

With permission from the Japan Agency of Cultural Affairs, we collected samples from wild individuals and from individuals kept in private houses, aquariums, and zoos throughout Japan from 2007 to 2015. We deposited voucher specimens in the Graduate School of Human and Environmental Studies, Kyoto University (KUHE) (Tables 1, 2, Fig. 1). Living Chinese giant salamanders and hybrids identified through genetic surveys were kept in aquariums and zoos for educational and scientific purposes. All experimental procedures in this study followed the experimental animal guidelines of Kyoto University and approved by the Ethics Committee for Human and Animal Research of the Graduate School of Human and Environmental Studies of Kyoto University (approval no. 29-A-7 and 30-A-7).

### Mitochondrial DNA (mtDNA) sequencing and phylogenetic analysis

We sequenced the mitochondrial cytochrome b (cyt b) genes of the Chinese giant salamanders and hybrids (detected by microsatellite analyses below noted: Table 1). To assign the samples collected in Japan into the genetic groups of Chinese giant salamanders, we compared these sequences with the sequences reported^4,8^.

We amplified a partial sequence of the cyt b gene region using PCR with the primers L13836 and H15297^14^ or HYD_Cytb_F1, HYD_Cytb_F2, HYD_Cytb_R1, and Salamander_Cytb_RN2^18^. The PCR products were sequenced with the PCR primers using the ABI 3130xl Genetic Analyzer (Applied Biosystems) and BigDye v3.1. We obtained sequences of *A. davidianus*, *A. jiangxiensis*, and *A. sligoi* from GenBank to identify each lineage (Table 1). Sequences were aligned using MAFFT^19^ with default settings. We conducted phylogenetic analyses using Bayesian inference (BI) methods. The most appropriate substitution model was selected based on the Bayesian information criterion using the Modeltest-NG program^20^. The BI tree was generated based on 10 million generations of Markov chain Monte Carlo runs using MrBayes v3.2.6^21^. We discarded the first 25% of generations as a burn-in segment, then sampled one of every 100 remaining generations. We verified the convergence of the Markov chain Monte Carlo runs using TRACER v1.6^22^. Posterior probability was used to assess the robustness of BI tree topology. We calculated the uncorrected p-distances of the partial cyt b gene between and within clades using MEGA v7.0^23^. The distance was calculated by pairwise deletion between samples with > 800 base pairs.

### Microsatellite analyses

To identify the introduced Chinese giant salamanders and hybrids, we used microsatellite markers developed for giant salamanders^24,25^. For reference data, we collected Japanese and Chinese giant salamanders in Japan (Table 2, Fig. 1).

We extracted genomic DNA from clipped tail fins using either a standard phenol–chloroform extraction procedure or a DNeasy Blood and Tissue Kit (Qiagen). We selected 14 microsatellite loci (AJP01, 03, 04, 06, 07, 07-2, 08, 08-2, 09, 11, 16, 26, 31, and AJ118^24,25^) and performed polymerase chain reaction (PCR) analyses, using the method of Yoshikawa et al.^25^. We measured PCR product size using the ABI PRISM 3130xl Genetic Analyzer (Applied Biosystems) and GeneMapper software (Applied Biosystems) with the GeneScan 500 LIZ size standard.

Based on the genotype, we inferred the hybrid class of each individual (pure *A. japonicus*, pure *A. davidianus*, F1, F2, backcross to each parental species) using NewHybrids^26^. We ran the software for one million sweeps after a burn-in period of 200,000 sweeps. For the analysis, we collected tissues from seven pure *A. davidianus* in captivity and 23 pure *A. japonicus* from Kyoto, Mie, and Nara Prefectures (Table 2). The *A. davidianus* sensu stricto individuals were directly obtained from the police or airports before 1990 and were identified as genetically pure (i.e., not hybridized individuals), although most of them have since died. The *A. japonicus* individuals were sampled from rivers in which no Chinese or hybridized individuals have been collected thus far. Morphological examination also supported the identification of these two species.

Each individual was classified into one of six categories: *A. japonicus*, *A. davidianus*, F1, F2, backcross with *A. japonicus*, and backcross with *A. davidianus*. We established the threshold for assignment of individuals to a category as a posterior probability of ≥ 0.8. Individuals with ambiguous ancestry were classified as “other hybrids”.

To survey overall genetic differences among samples of pure *A. davidianus*, *A. japonicus*, *A. sligoi*, and U1 lineage, which were identified by mitochondrial phylogenetic analysis and NewHybrids analysis, we performed principal component analysis (PCA) using genotype information obtained from microsatellite analyses. We performed PCA using GenoDive^27^.

### Genetic sex identification

Sexing giant salamanders based on external characteristics alone is difficult, except during the breeding season. Therefore, we conducted genetic sexing of live *A. sligoi*. Female-specific genetic markers (i.e., adf225, adf318, adf340, and adf431) were used in accordance with the method of Hu et al.^11^ to characterize two live giant salamanders identified as *A. sligoi*. One of the three *A. sligoi* individuals that had been kept in a private house in Okayama Prefecture likely died in 2020 (about 1100 mm in total length estimated from a photo), and no voucher specimen was collected. Additionally, two male and two female specimens of *A. davidianus* (KUHE 47438, 56335, 58902, and 58903) were sexed via direct gonad observation.

### Morphological examination of *A. sligoi*

Living *A. sligoi* specimens were photographed to document their body shape and coloration; their total lengths were measured using a tape measure. Because of the risk of physical harm to the animals, we refrained from detailed examination of their morphology. One voucher specimen of *A. sligoi* preserved in Kyoto University (KUHE 41444) was measured in accordance with the method of Hara et al.^28^, and we also measured all fingers and toes. Tubercles on the ventral surface were also confirmed. The body coloration was compared with descriptions by Boulenger^12^ and the illustration in Fig. 2 of the work by Turvey et al.^8^.

### Supplementary Information

Supplementary Information.

## Data Availability

The newly obtained sequences and microsatellite data are deposited in GenBank (cyt b sequence: accession nos. LC650372–LC650454, LC728249) and in figshare (microsatellite: https://figshare.com/search?q=10.6084%2Fm9.figshare.24211173).
